# Q and A What is a pathogen? A question that begs the point

**DOI:** 10.1186/1741-7007-10-6

**Published:** 2012-01-31

**Authors:** Liise-anne Pirofski, Arturo Casadevall

**Affiliations:** 1 Departments of Microbiology and Immunology and Medicine (Division of Infectious Diseases) of the Albert Einstein College of Medicine and Montefiore Medical Center, 1300 Morris Park Ave, Bronx, NY 10461, USA

## What is a pathogen?

A pathogen is usually defined as a microorganism that causes, or can cause, disease. We have defined a pathogen as a microbe that can cause damage in a host. However, this definition immediately raises the question of what it is about the microorganism that enables it to cause disease or damage; and this takes us to an ongoing debate that dates back to the late 19th century when the germ theory of disease was established. In the early days of the germ theory era many of the major pathogenic microbes were encapsulated or toxigenic bacteria, and this suggested that there were inherent differences between pathogenic and non-pathogenic microbes. However, even then it was obvious that neat classifications were problematic, for it was known that a microbe could be attenuated in the laboratory, but virulence could be restored by passage in a host, suggesting that the same microbe could exist in pathogenic and non-pathogenic states.

## Hang on - surely encapsulation and toxin production are inherent properties of the microorganism, so doesn't the fact that these properties can be lost just mean pathogens can become non-pathogens?

Yes. But it's more complicated than that. First of all, a factor or product that confers pathogenicity in a normal host cannot be identified for many microbes. Second, properties conferring pathogenicity depend as much on the host as they do on the microorganism: encapsulated bacteria are pathogenic because they have a polysaccharide coat that prevents phagocytic cells from seeing them, and thereby avoid immediate elimination by the innate immune system of the host. Even toxins are damaging because they disrupt essential host functions. However, it was developments in the 20th century that clearly obliterated the hope of ever drawing a clear and unequivocal line of distinction between pathogens and non-pathogens. Beginning in the 1950s the introduction of broad spectrum antimicrobial agents, immunosuppressive therapies, newer types of surgery, including organ transplantation and joint replacement, implantable devices and indwelling catheters, each of which alters host-microbe interactions, turned out to create conditions in which the host became vulnerable to microbes that were previously considered non-pathogenic. As a result, it became apparent that many microbes previously considered non-pathogenic, or rarely pathogenic, such as *Staphylococcus epidermis *and *Candida albicans*, could cause serious disease.

## I can see how immunosuppression could make you vulnerable to the damage that microbes can cause, but antibiotics? Surgery?

Right. Antibiotics make people more vulnerable to microbe-mediated damage because they alter the microbiota, or the normal microbial flora, and the balanced relationships between the microbes that reside in the mucosal niches in the body and the host structures that support these communities. Surgery can have the same effect by removing or altering normal mucosal and cutaneous barriers to infection. So the effects of antibiotics and surgery enhance the pathogenicity of microbes that do not ordinarily cause damage or disease in normal microbial communities, or intact mucosal and cutaneous surfaces, by making the host more susceptible to damage or invasion.

## So pathogenicity can depend on whether some artificial situation enables the microorganism to infect the individual?

In part. Many microbes cause disease in some, but not all of those individuals who are infected with them. In fact, many microbes that cause disease are already present in the individual and the individual is thus already 'infected'. This is exemplified by microbes such as staphylococci and *Candida *spp., which are actually present in most individuals, but only cause disease in some. This also applies to many other microbes, including those to which an individual is immune, either through prior infection or through vaccination, as immune individuals are recognized as being resistant to the capacity of a microbe to cause disease.

## But surely in the case of immunity the pathogen is still a pathogen, it's just that immunity prevents you from getting sick, right?

Not really. The question implies that the ability to cause damage or disease is an inherent microbial property, but in fact these characteristics only exist in the context of a susceptible host. Therefore, when a host is immune, pathogenicity is not expressed. What is important to recognize is that pathogenicity and virulence are microbial properties that can only be expressed in a susceptible host.

## What about the bacteria that normally inhabit our gut without causing disease - the so-called commensal bacteria: how does the immune system distinguish these bacteria from pathogens?

The immune system does not distinguish between pathogens and commensals. In fact, the question of whether pathogenicity is a microbial trait and the question of whether hosts distinguish so-called pathogens from non-pathogens have the same answer: pathogenicity is an outcome of host-microbe interaction and is thus inextricably linked to characteristics of the host as well as those of the microbe. Rather than distinguishing commensals from pathogens/non-pathogens, the immune system of healthy hosts actually depends on these microbes. Commensals (also called the microbiota) are acquired by infection soon after birth, after which they establish residence in mucosal niches where they replicate, and there is increasing evidence that the microbiota play a crucial role in the development of the immune system and that the immune response to the bacteria in mucosal niches helps maintain barriers to invasion on surfaces exposed to potentially harmful microorganisms. The commensal bacteria themselves do no harm, provided that the immune system and mucosal barriers remain normal and intact. The immune system provides a large variety of tools - cells and molecules - that recognize, react to and control microbial growth and invasion, often in a manner that does not result in host damage or disease, and when this happens, there is no readout. In this instance, the immune system might be thought to have distinguished a pathogen from a non-pathogen, but in fact, it simply controls microbial growth and/or invasion in a manner that does not translate into microbial pathogenicity.

In a situation where there is host damage or disease, there are two possibilities: either the immune system did not contain or control the microbe and the microbe caused host damage, or the host immune response to the microbe caused damage or disease, whether the microbe was controlled, or contained, or not. Thus, the immune system does not discriminate between microbes; it reacts to them, albeit differently depending on characteristics of the host and characteristics of the microbe, with the response defining an outcome that reflects the behavior of host and microbial factors.

## So pathogenicity can be due to the immune response to the pathogen rather than the pathogen itself?

Absolutely. The obvious case is where the immune response to some microbe is insufficient, and the microbe can replicate and disseminate throughout the host. In this instance, the lack of an immune response translates into the potential for pathogenicity (as mentioned above, even commensal bacteria can be pathogenic if the immune system is impaired or the mucosal barrier is disrupted). This can occur because of the lack of a cellular or secreted factor that is needed to contain or control the microbe, and/or host or microbial factors that enable the microbe to evade the host response. An interesting paradox occurs in the case of two bacteria that produce toxins generally regarded as factors increasing the virulence of the microbe**: s**taphylococci that produce a so-called leukocidin, and pneumococci that produce a toxin called pneumolysin. Because these toxins also activate the innate immune response, bacteria that do not produce them can sometimes be more pathogenic than bacteria that do. Thus, when the immune response to a microbe is insufficient, microbial factors can cause damage, and when microbial factors fail to stimulate the immune system, the microbe can disseminate and cause disease.

At the other end of the spectrum, when the immune response to a microbe is too exuberant, it can be the immune response itself that is responsible for the pathology. When damage occurs in this setting, it is most commonly due to detrimental inflammation and can occur whether the microbe is controlled or contained or not. Examples of this phenomenon include diseases like toxic shock syndrome, in which it is the potent activation of the immune response by a microbial component that does the damage. In these diseases, antimicrobial therapy is often unsuccessful because it does not reduce the host inflammatory response. In fact, new directions in the treatment of infectious diseases that are marked by exuberant inflammation increasingly involve the use of anti-inflammatory therapies.

## You mentioned that the toxins produced by staphylococci and pneumococci increase their virulence - what is the difference between pathogenicity and virulence?

Although these terms are often used interchangeably, they have different meanings [6]. Pathogenicity is defined by the capacity of a microbe to cause damage in a (susceptible) host. Virulence is defined as the relative capacity of a microbe to cause damage in a host. Although both pathogenicity and virulence can only be manifest in a susceptible host, pathogenicity is a discontinuous variable, that is, there is or is not pathogenicity, whereas virulence is a continuous variable, that is, it is defined by the amount of damage or disease that is manifest. Virulence is a relative term for there is no absolute measure of virulence and virulence is always measured relative to another microorganism (for example, an attenuated strain, or a different species). Although they differ as delineated here, pathogenicity and virulence are both microbial variables that can only be expressed in a susceptible host, underscoring that each is dependent on host variables.

## What is the difference between an opportunistic pathogen and any other kind of pathogen?

There is no difference between an opportunistic pathogen and any other kind of pathogen. Both are microbes and both have the potential to cause damage/disease in a host. The definition that is often used for opportunistic pathogens is that these microbes cause disease in people with impaired immunity but not in normal individuals. However, this definition is purely operational: the same microbe - consider *Candida albicans *and *Staphylococcus epidermidis *- can cause disease in one individual but live harmlessly in others, which means that the same microbe would be called an opportunist in one individual and a commensal in another. Indeed, the identification of certain microbes as a cause of disease in certain hosts can unmask or be a sentinel for an underlying immunodeficiency.

However, although the absence of certain host factors or products can lead to an inability to control or contain certain microbes, the determinants of pathogenicity and virulence for these microbes depend on host and microbial factors, as is the case for all microbes. In our view there are only microbes and hosts and the outcomes of their interactions, which include commensalism, colonization, latency and disease. Hence, attempts to classify microbes as pathogens, non-pathogens, opportunists, commensals and so forth are misguided because they attribute a property to the microbe that is instead a function of the host, the microbe, and their interaction.

## Can the emergence of new pathogens be predicted?

Yes and no. Pathogenicity and virulence are emergent properties, meaning that they cannot be predicted directly from the properties of the microorganism. The environment, an individual host or population of hosts and/or an individual microbe or population of microbes can change independently, or as a function of complex interactions, including those between environment and host, host and microbe, microbe and environment, and all three. Thus, microbial pathogenicity is intrinsically unpredictable. Host and microbial characteristics are subject to predictable and unpredictable changes prompted by known, unknown, and random environmental, immunological, and other factors. Thus, as it is an outcome of host-microbe interaction whereby each entity is subject to independent and dependent changes at any point in time, pathogenicity is an emergent property.

## So is prediction hopeless?

Not altogether. It is possible to test predictive hypotheses on microbial pathogenicity in model systems in which microbe and/or host can be held constant. That said, however, neither the complexity nor the variability or randomness that occurs in nature occurs or can be recapitulated in models systems. Thus, while predictions on how given (known) variables might affect the potential for a (new) microbe to be pathogenic in a given (known) population might be possible, such predictions are only possible in the context of available knowledge and paradigms. This being the case, prediction of the emergence of new microbes with the potential for pathogenicity will always be subject to severe limitations. Clearly, the continued acquisition of new knowledge and development of new scientific and intellectual platforms and paradigms will be important in bringing our models closer to reality.

## Where can I find out more?

Armstrong D: **History of opportunistic infection in the immunocompromised host**. *Clin Infect Dis *1993, **17(suppl):**S318-S321.

Casadevall A, Pirofski L: **Host-pathogen interactions. II. The basic concepts of microbial commensalism, colonization, infection and disease**. *Infect Immun *2000, **68:**6511-6518.

Casadevall A, Pirofski L: **Host-pathogen interactions: the attributes of virulence**. *J Infect Dis *2001, **184:**337-345.

Casadevall A, Pirofski L: **The damage-response framework of microbial pathogenesis**. *Nat Rev Microbiol *2003, **1:**17-24.

Casadevall A, Fang FC, Pirofski LA: **Microbial virulence as an emergent property: consequences and opportunities**. *PLoS Pathog *2011, **7:**e1002136.

Coleman JR, Papamichail D, Yano M, Garcia-Suarez MM, Pirofski LA: **Designed reduction of Streptococcus pneumoniae pathogenicity via synthetic changes in virulence factor codon-pair bias**. *J Infect Dis *2011, **203**:1264-1273.

Corrales-Medina VF, Musher DM: **Immunomodulatory agents in the treatment of community-acquired pneumonia: a systematic review**. *J Infect *2011, **63:**187-199.

Vandermeer ML, Thomas AR, Kamimoto L, Reingold A, Gershman K, Meek J, Farley MM, Ryan P, Lynfield R, Baumbach J, Schaffner W, Bennett N, Zansky S: **Association between use of statins and mortality among patients hospitalized with laboratory-confirmed influenza virus infections: a multistate study**. *J Infect Dis *2012, **205:**13-19.

Yoong P, Pier GB: **Antibody-mediated enhancement of community-acquired methicillin-resistant Staphylococcus aureus infection**. *Proc Natl Acad Sci U S A* 2010, **107:**2241-2246.

